# An Impact Factor for Tropical Medicine and Infectious Disease

**DOI:** 10.3390/tropicalmed7070140

**Published:** 2022-07-20

**Authors:** Peter A. Leggat, John Frean

**Affiliations:** 1 College of Public Health, Medical and Veterinary Sciences, James Cook University, Townsville, QLD 4811, Australia; 2 Centre for Emerging Zoonotic and Parasitic Diseases, National Institute for Communicable Diseases, Johannesburg 2131, South Africa; johnf@nicd.ac.za

*Tropical Medicine and Infectious Disease* (TMID or TropicalMed) was established in 2016 [1] as the Official Publication of The Australasian College of Tropical Medicine [2] and published by the Multidisciplinary Digital Publishing Institute (MDPI) based in Switzerland and many other countries [3], being one of the oldest online publishers still existing after 25 years. It was founded with an international editorial board that continues to expand and organize under the leadership of A/Professor John Frean, Editor-in-Chief, from the National Institute for Communicable Diseases and Wits Research Institute for Malaria, University of the Witwatersrand, South Africa, and Professor Peter A Leggat, Deputy Editor-in-Chief, from James Cook University, Australia. It is indexed by all of the major indexing and abstracting services, including PubMed and Web of Science. Hence, it is tracked for impact factor and recently received its first one, a very creditable 3.711. The definition of the impact factor is as follows:

*“The definition of impact factor is the number of citations in the given year, for the articles published in that journal during the last two preceding years, divided by the total number of citable items, which were published in that journal in the previous two years.”*

[4]

An impact factor of this magnitude, in the medium or good category, for a first impact factor, is a credit to all the researchers supporting the MDPI open access platform and authors of general manuscripts and work specific for the various Special Issues of the journal. TMID now has one of the highest impact factors in the field of tropical medicine, ranking 6th out of 24 titles (Q1); the journal ranks 11th out of 39 titles (Q2) in the category of ‘Parasitology’, and 59th out of 94 titles (Q3) in the category of ‘Infectious Disease’. It is a wonderful achievement for MDPI and the TMID Managing Editor, Beryl Liu and Publishing Manager, Peter Roth, particularly in promoting informative Special Issues and developing an efficient review and publication cycle.

Having an official impact factor will make *Tropical Medicine and Infectious Diseases* very attractive to authors and institutions, and the editorial staff can anticipate an increase in the number of manuscripts submitted. We hope it will also focus attention on The Australasian College of Tropical Medicine, and help it to advance its mission in the field of tropical medicine. 
